# Application of indirect observation within mixed methods to contrast the results obtained through direct observation: a discussion with observed Pikler educators

**DOI:** 10.3389/fpsyg.2024.1401535

**Published:** 2024-06-26

**Authors:** Haizea Belza, Jone Sagastui, Elena Herrán

**Affiliations:** ^1^Department of Education Sciences, University of the Basque Country UPV/EHU, Bilbao, Spain; ^2^Department of Language and Literature, University of the Basque Country UPV/EHU, Bilbao, Spain; ^3^Department of Developmental and Educational Psychology, University of the Basque Country UPV/EHU, Bilbao, Spain

**Keywords:** early education, mixed methods, indirect observation, in-depth interview, Pikler-Lóczy educational approach

## Abstract

**Introduction:**

This work arises from a previous research, “Pikler educators early in the morning” carried out in the Emmi Pikler Nursery School in Budapest through Systematic Observation. In it, Piklerian choreographies were found in observed educators’ behavior during the studied three daily activities: feeding breakfast, dressing to go to the garden and free play accompanying. All of them share certain Piklerian principles, which are synthesized in three central keys: the stability of the educator’s behavior, her strategic and intentional positioning, and an active emotional control.

**Objectives:**

This study aims to contrast this synthesis of results by means of an in-depth interview with the two observed educators, and to apply the methodological approach of indirect observation within mixed methods for its analysis. The objective is to confirm whether the three central keys are recognized as their own and to look for new theoretical-practical elements within the studied educational approach.

**Materials and methods:**

We applied an in-depth interview and analyzed it following the guidelines of indirect observation. The participants were the two educators previously observed, a translator from the Pikler team, and the three observers, authors of this work. An *ad hoc* observation instrument was elaborated, and the three macro-stages QUAL-QUAL-QUAL proposed within mixed methods were rigorously followed.

**Results:**

Lag sequential analysis was used to conduct data analyses. We deepened in prospective lags and obtained the response pattern underlying the interview. Then, we performed a concurrence analysis to investigate the relationship between the central keys obtained in our original research and Piklerian ideas.

**Conclusion:**

In-depth interview within mixed methods has been a novel and generous tool leading us to substantial and methodological contributions, despite the simplicity of performed analyses. Interviewed educators’ response pattern is a faithful reflection of the Piklerian modus operandi. The study of concurrences shows that Piklerian education is something natural, integrated in its professionals, with the exception of emotional control, which still requires permanent reflection.

## 1 Introduction

The baseline of the present paper are the results obtained in the research “Pikler educators early in the morning” (Belza, 2020; Belasko, 2023; Sagastui, 2023) in which Observational Methodology (OM) (Anguera, 1979) was applied to study daily interactive routines at the Emmi Pikler Nursery School in Budapest. Specifically, the aforementioned research focused on everything that happened in the studied educational center during the morning, so educators’ four distinct activities were identified and investigated: feeding breakfast, changing diapers, dressing children to play outside and accompanying their free play. The study of educators’ behavior both in body cares (Belza, 2020; Belasko, 2023) and while they accompanied children’s autonomous activity and free play (Sagastui, 2023) led to valuable conclusions about quality early education. This was possible thanks to the perfect fit between the studied educational approach and the characteristics of OM (Anguera, 2003), a methodology within mixed methods. Now, we intend to go one step further and reaffirm the obtained results, in this case, by using indirect observation; more specifically, by conducting an in-depth interview with the experienced educators observed in the original research.

Pikler-Lóczy education is an early and quality educational approach that emerged in Budapest in 1946, when pediatrician Emmi Pikler and her team took over a foster home in Lóczy street, from which it took its former name: Lóczy Institute or Lóczy foster home (Falk, 2018c). Since then and up to the present day, this center has changed its name multiple times —Methodological and Pedagogical Institute for the Care of Babies and Young Children, Emmi Pikler National Institute of Pedagogical Methodology of Foster Homes—, and has become a European reference in quality early childhood education, continuous professional training in this educational stage, and rigorous scientific research, a transcendent endorsement of its particular way of doing things (Herrán, 2013; Herrán, 2014). In 2006 the original center —the foster home— closed and Emmi Pikler Nursery School opened its doors in the same location.

The founder’s previous training as a pediatrician was key in her subsequent professional journey. In fact, she learnt almost every principle that marked her as a doctor during her studies in medicine, which she first applied in her own family and then professionally, as a family pediatrician: to observe the child’s body in its natural environment; to conceive physical and psychological development as inseparable and intimately conditioned by the physical and human conditions of the context (Wallon, 1980, 1985); to understand prevention as the establishment of healthy living and developing conditions; to respect the child’s characteristics; to promote children’s activity and, to that end, to create *ad hoc* play spaces and provide children with clothing that would not restrict their movement; to seek children’s cooperation all the time, even in medical check-ups; to safeguard children’s autonomous motor development as the basis for healthy, progressive and safe movements, which were not observed among children who were taught to move; and a long *etcetera*. After verifying the model’s viability, the challenge was to find the precise conditions for a healthy and balanced education in an institutional, collective context as Lóczy foster home (Pikler, 1969; Herrán, 2013; Falk, 2018c).

This was certainly an ambitious and accurate model since it aimed to generalize family upbringing to the collective context of the foster home. To this end, the conditions surrounding babies and young children were closely reflected on; the goal was to establish a comprehensive early education system, focused on babies’ and young children’s characteristics, and to investigate their particular *modus operandi*, mainly through longitudinal observations of child development and its conditions. The result was a complete success (Pikler, 1968, 1969). The guiding principles of this approach are (David and Appell, 1986, 2010; Falk, 2008): the importance of a privileged affective professional bond, differentiated from the family bond, since the relationships between babies and their caregivers at the foster home were not familiar; the value of young children’s autonomous activity, so that the resulting motor development is harmonious and can lay the foundations for a promising intellectual development; the convenience of promoting children’s awareness, both of themselves and of their environment; and the importance of a good state of health, origin and result of the correct application of the previous principles.

It is worth adding that, within this educational approach, some central issues can be highlighted, such as the areas of early development (Belza et al., 2019, 2020) or the intervention modalities that accompany them (Sagastui et al., 2022). Among the areas of early development, on the one hand, central and some other punctual body cares can be distinguished: from diaper changing (Falk and Vincze, 2018) or feeding (Vincze, 2018), to wiping the nose, or adjusting the child’s clothes (Hevesi, 2018). On the other hand, there is autonomous activity: from movement itself and the postural development associated with it (Pikler, 1969), to manipulation with one’s hand and, with it, infant play (Kálló and Balog, 2013; Tardos, 2014). Within these, there are some activities in which the infant needs direct adult intervention and some others that he or she can perform on his or her own either with indirect help or without it, but always with the adult’s presence or supervision (David and Appell, 1986, 2010; Falk, 2018d; Tardos, 2008, 2014; Szöke, 2016). Overall, every activity pursues the same goal: the voluntary and happy progression of children’s autonomous behavior.

In order to consolidate this educational approach, meticulous self-research was essential. In fact, the model was developed through the application of their own modality of systematic perception, known as Piklerian observation (Mózes, 2016; Tardos, 2018b). This comprehensive research enabled the team to deepen in their knowledge on early development and about their intervention or educator-child interaction and, at the same time, to improve their professional practice. To achieve this goal, they had to put a large number of factors under control, both of form and substance (Mózes, 2016). In relation to the former, the observation they conduct to study their very educational approach is direct, focuses on everyday situations of the natural context, and both the recording and the treatment of data are rigorous, contrasted, discussed and reworked at three levels: interpretative, explanatory and propositional (Mózes, 2016; Tardos, 2018b). In relation to the latter, it is important to highlight that, in seeking to describe the manifestations and behavior of children as well as the most appropriate conditions for intervention as concretely and rigorously as possible, they have developed a common terminology throughout their history. It is a system of well-defined and shared concepts and vocabulary that not only objectifies what is observed, but also gives it a greater degree of temporal and spatial stability, standardization of the framework and certainty in the framing, which makes it possible to conduct better comparisons and analyses than other observational modalities (Mózes, 2016, Tardos, 2018b).

Thus, beyond organizational issues (David and Appell, 1986, 2010; Tardos and David, 2018), such as having a small number of children per classroom, distributed among the three educators of the group —so that each one is responsible for a small number of children, three or four at most, in order to be able to take care of them in good conditions (Falk, 2018a)—, or a precise agenda —regular but adaptable to the circumstances of everyday life with all its vicissitudes—, the educational activity as a whole requires a careful preparation of its human and material conditions (Wallon, 1978). Pikler educators conduct each and every body care in such a way that the infant is the protagonist, the center of their action. So everything the educator does or proposes to the infant is done in practically the same order each and every time (Herrán, 2013; Falk, 2018b), and at the pace that this infant requires, while simultaneously and permanently taking into account and attending to the infant’s varied gestures, actions, responses, comments, etc., encouraging his or her participation (Hevesi, 2018), awareness and, with them, their autonomy (Falk, 2018d). Novel educators receive specific initial training in addition to monitoring by their experienced colleagues, so that they progressively internalize the Piklerian *modus operandi* (Kelemen, 2016). All educators share what they observe on a daily basis, so that they have a deep knowledge of each of the babies or children in the group (Mózes, 2016; Tardos, 2018b). The spaces, materials, objects and toys both in the classroom and in the garden, where they spend most of their time, are thoughtfully selected and organized, responding to infants’ current interests, preferences and capabilities (Kálló and Balog, 2013; Tardos, 2014, 2018a).

Regarding body cares, this preparation permits the educator to be able to fully focus on the care itself, and on each child, his or her pace, ability and interest. All of them are developed following a precise choreography, which all educators share (Herrán, 2013; Falk, 2018b). This choreography is the way to homogenize all the interventions with each child, thereby emulating the family environment. On the other hand, autonomous activity has a great place in infants’ life; at the beginning, free movement and play take place simultaneously, and these immediately differentiate from each other, so that their conditions do too (Kálló and Balog, 2013; Tardos, 2014; Tardos and Szanto-Feder, 2018). That is why, in addition to having two square meters per child in the classroom —so they can move freely as they conquer the vertical posture—, one of the structures that favors motor skills invented by Emmi Pikler herself is incorporated in the center of the classroom, such as the Pikler labyrinth, the Pikler triangle with its ramp, or platforms of various sizes (Pikler, 1968; Falk, 2018d). In relation to free play, the material they offer is varied and always appropriate to children’s developmental level, preferences and interests: from a dotted cotton cloth with bright and contrasting colors that is given from the moment the baby is able to differentiate his or her own hand, to books or more complex didactic games (Kálló and Balog, 2013; Tardos, 2014; Szöke, 2016).

The Emmi Pikler Nursery School is the faithful heir of all the knowledge accumulated during the 65 years of the Lóczy foster home’s history, and it is where the original research referred to in this article —initiated by Herrán in 2013 (Herrán, 2013; Belza et al., 2023b)— was carried out. Unlike the usual Piklerian research, which mainly observed child development, and basically due to temporal issues, its object of study was the activity of the Pikler educator or choreography. Thus, the objective was to empirically demonstrate the Piklerian choreographies deployed in each area of early development and, secondarily, to unravel their educational potential (Belasko et al., 2019; Belza et al., 2019; Sagastui et al., 2020). The systematic observation of two experienced educators early in the morning while they attended to and accompanied the children in their respective groups over a period of 3 months allowed a comprehensive study of the updated Pikler-Lóczy educational approach. Thus, OM (Anguera, 1979, 2003) turned out to be the ideal methodological option for the proposed objective, as it allows to capture spontaneous behavior in its natural context, deconstruct it and analyze it to obtain a pattern that, in this case, was expected to fit with the studied Piklerian choreographies, nuanced and previously demonstrated by the Pikler team itself.

OM provides guidelines and valuable procedural resources to combine qualitative and quantitative perspectives along the same research process, which led it to be considered a mixed method itself (Anguera et al., 2018a; Hitchcock and Onwuegbuzie, 2022).

In this sense, the proposal made from OM is based on the ordered succession of QUAL-QUAN-QUAL stages (Anguera and Izquierdo, 2006; Sánchez-Algarra and Anguera, 2013). The first, qualitative, corresponds to the development of the observational instrument (Anguera et al., 2007) and, with it, the systematic recording of all the units extracted from the reality to analyze (Anguera and Blanco-Villaseñor, 2003). The sample collected in the original research referred to in this study reports on three types of body care —changing diapers, feeding breakfast and dressing children to go out to the garden— and on free play accompanying (Belza, 2020; Belasko, 2023; Sagastui, 2023). The *ad hoc* observational instruments used in these investigations incorporate a common part, related to the shared Piklerian *modus operandi*, and a specific part, associated with the daily moment studied in each case and its conditions.

Precisely, the systematic recording conducted through the observation instrument makes it possible to systematize the initially descriptive recording in a code matrix that can be analyzed through specific quantitative techniques for categorical data (Anguera et al., 2021). Thus, OM relies on the *connect* option —proposed by Creswell and Plano Clark (2011) as one of the three possible ways to carry out mixing within mixed methods research: merge, connect, embed—, since this option allows to conduct the *quantitizing* (Sandelowski et al., 2009), which, in this case, is carried out with the obtained observational data. In other words, the second stage of the research process, aimed at ensuring the rigor of the recording through the quality data analysis (Blanco-Villaseñor and Anguera, 2003) and analyzing of the collected observational data itself (Blanco-Villaseñor et al., 2003), is characterized by being predominantly quantitative.

Considering the general objective, the demonstration of the choreographies displayed by Pikler educators, and the specific ones —which included obtaining the behavioral patterns of each body care or moment, the study of their different parts and intervention modalities, the comparison between both educators in search of a shared *modus operandi*, etc.— data analysis techniques were different in each research. The proposal made through OM does not combine methods or techniques, nor data from different sources, but transforms the collected qualitative information into data susceptible to analysis with appropriate quantitative techniques (Anguera et al., 2020). Specifically, three techniques were used in the original research: lag sequential analysis (Bakeman, 1978; Bakeman and Quera, 2011), polar coordinate analysis (Sackett, 1980; Anguera, 1997) and T-Pattern detection and analysis (Magnusson, 1996, 2000, 2020). These techniques are based on the primary parameters of order and duration, which include frequency too. According to several authors, this makes the integration more robust than most empirical publications on mixed methods that only apply conventional analytical techniques based on frequency (Anguera et al., 2020). Moreover, it is worth mentioning that a number of studies within the educational field have started to use this methodological approach (e.g., Bonilla et al., 2020; Escolano-Pérez et al., 2020; Navarro-Adelantado and Pic, 2021; Pic et al., 2021).

The third and final stage, qualitative, corresponds to the interpretation of the results in relation to the objectives of the study and, in this case, Piklerian theory (Anguera et al., 2018b). Thus, the studies conducted on giving breakfast (Belza et al., 2019, 2020, 2023a,b; Belza, 2020), dressing to go out to the garden (Belasko et al., 2019, 2022, 2023; Belasko, 2023) and free play accompanying (Sagastui et al., 2020, 2022, 2023, 2024; Sagastui, 2023) bring together some basic Piklerian ideas that, indeed, emerge from the analyzed daily educational practice, intimately related to each other. These are, among others, the methodical empathic adoption of the child’s perspective; a certain perspective taking or emotional distance, which allows an important degree of objectification when analyzing habitual situations; placing personal aspects under control, based on the internalization of a shared paidocentric or child-centered educational culture; the coherence and support of the educational team, which reflects together, periodically and systematically; the importance of daily observations and their analysis and elaboration, in view of the individual adjustments to each child; the heritage of the foster home, partly evident in the organization and functioning of the nursery school, and also latent in subtle educational attitudes that optimize the way of educating, ensuring its quality.

Among the results obtained in the original research, three central keys arose. Namely, the *stability* of Pikler educators’ behavior, always adaptable to the human and physical circumstances of each moment; their *strategic and intentional positioning*, which allows the optimal focus of attention and the ergonomics of the intervention; and an active and fluid *emotional control*, associated to professional empathy, to the children’s protagonism in their own education, as well as to the awareness of being a permanent role model. At this point, beyond being able to go deeper into each daily activity, it was deemed necessary to return to Lóczy in order to contrast the obtained results, and check if they really reflect the perspective of the studied educators.

This line of research has provided new and enlightening knowledge about Pikler-Lóczy education thanks to the use of OM, as a mixed method; since, in the third stage of the process, the behavior of the observed educators was not only described, but also explained. This task has been exceptionally simple because of the complementarity between observational methodology and the Pikler-Lóczy educational approach, since this very model is defined by its own, characteristic and original observational tradition. In fact, Piklerian observation supported the development of the choreographies of daily educational tasks and permitted to qualify, clarify and model them observation by observation, child by child, care by care, definition by definition, until they were constituted, thanks to the quality and rigorousness of their continuous work and effort.

Now, the aim is to go one step further and discuss the interpretations of the behavioral patterns found in the original research, “Pikler educators early in the morning” together with the observed experienced educators. Thus, we used indirect observation (Anguera et al., 2018c; Del Giacco et al., 2019, 2020) to meet this goal, specifically, an in-depth interview (Anguera, 2021). So, after several face-to-face and formal meetings with the educational team in Lóczy to present them the results of the original research, a virtual meeting was arranged between the researchers and the educators, supported by a translator, aiming to exchange opinions on the obtained results. This way, we close the research with this third stage of OM, as we go back to the observed educators, so that they can confirm or refuse the conclusions about their own practice, validating, if necessary, the conducted research.

Therefore, the general objective was to contrast certain central results obtained in the original research, “Pikler educators early in the morning” by conducting an in-depth interview, a modality of indirect observation within mixed methods, to the two educators observed in it, and in doing so, to clarify three key ideas. This general objective unfolded two specific objectives. The first was to confirm whether, in the expert opinion of the observed educators, the three central keys found in the original research exist in their daily practice and in their shared reflection, and if so, whether they recognize them as their own. The second was, based on this potential recognition, to trace new theoretical-practical elements that may qualify, broaden or enrich the Piklerian educational discourse.

## 2 Materials and methods

This work was developed using Observational Methodology (OM). OM represents the appropriate methodological approach to study and evaluate spontaneous behavior in its habitual context (Anguera, 1979, 2003), and it is a field that has gone through great advances at a procedural, technological and also conceptual level. So much so that it is now being used as a suitable approach for the analysis of in-depth interviews.

Following the three macro-stages QUAL-QUAN-QUAL proposed by mixed methods (Anguera, 2021), the transcription of the in-depth interview (QUAL stage) was the starting point for quantitizing. Based on the corresponding theoretical framework and a certain empirical expertise, an observation instrument was constructed with a defined structure. This instrument made it possible to systematize the transcription until it took the form of a code matrix, which depicts information that was still qualitative although already highly systematized. These data could be later subjected to quantitative analyses appropriate for categorical data (QUAL stage). Through this procedure it was possible to detect the underlying structure of the in-depth interview, as well as to ascertain the map of relationships between codes/sub-dimensions revealed by the results of the analysis and interpreted by returning to the theoretical framework (QUAL stage).

### 2.1 Participants

The interview was conducted with two expert educators of the Emmi Pikler Nursery School, currently active and with more than 30 years of experience in this institution. It is worth mentioning that the team of professionals of this educational center includes different profiles, from pedagogues to pediatricians, educators, psychologists, etc. However, as the object of study of our original research was the behavior of these educators in their own classroom, educators were considered the most suitable professionals to achieve the objective of this work, which was to analyze the discourse about their own practice.

In this sense, in this nursery school there are a total of three groups of children, and three educators for each of them, a total of nine. The criteria for selecting the educators to be interviewed were: (1) having completed their professional training in Lóczy’s own nursery school under the direction of pediatrician Emmi Pikler; (2) having actively participated in the continued professional development programs planned by the institution; (3) being familiar with the results of our original research about their own educational practice as a result of having attended the research feedback sessions held in September 2023 in Lóczy.

After applying the aforementioned criteria, two educators were selected, in addition to a translator from the institution itself and therefore also an expert on the object of study. A 47-minute in-depth interview was conducted. In addition to the two educators and the translator, the three authors of this paper participated in the interview. One of them was the interviewer, while a second one supported her and the third one acted as a listener.

### 2.2 Instruments

An observation instrument was developed *ad hoc* to analyze the in-depth interview. Precisely, we created a field format because it is more flexible and allows the study of several levels of response simultaneously (Anguera et al., 2007). The dimensions to include within the observational instrument were proposed based on the content of the in-depth interview questions and were supported by the theoretical framework, which despite not being required for a field format development, it is advisable (Anguera et al., 2018a). In this case, the instrument includes four dimensions, in addition to the dimension that serves to identify the speaker of each recorded textual unit. The first dimension refers to the sections of the interview, mainly divided into the three central keys found in the original research and previously presented. The second dimension specifies the communicative intention of the interlocutor in each textual unit, so it is divided into two sub-dimensions, one referring to the interviewer, and the other to the interviewees. The third dimension details the content of the interview, specifically, the ideas that the educators express in their discourse or about their own practice. Finally, the fourth dimension groups together paralinguistic behaviors, considered relevant given the frequent use of gestures and non-linguistic cues by participant educators.

Once the dimensions to examine are proposed, the development of an *ad hoc* instrument requires repeated exchanges between the theoretical framework and the obtained responses, aiming to establish a catalog of codes or diverse behaviors in each dimension, which must be mutually exclusive (Anguera et al., 2018a).

Table 1 lists the dimensions, sub-dimensions and codes included in the field format.

**Table 1:** Field format.

Dimension	Sub-Dimension	Code
Participant	Interviewer 1	E
	Interviewer 2	HB
	Translator	EM
	Interviewee 1	JK
	Interviewee 2	JH
	The three of them together	LT
Section of the interview	Other: previous moments, greetings, etc.	A0
	(1) Stability/adaptability	A1
	(2) Focus of attention, strategic positioning	A2
	(3) Emotional control, acting as a role model	A3
	End of interview and giving thanks	A4
Communicative intention	Others: idiomatic, technical, etc.	I0
Interviewer	Asks	I11
	Explains, raises point	I12
	Reformulates to clarify idea	I13
	Reformulates to confirm she has understood	I14
	Confirms	I15
	Asks translation	I16
	Denies	I17
Interviewee	Confirms	I21
	Adds new information	I22
	Exemplifies	I23
	Reformulates in the same sense	I24
	Reformulates in another sense	I25
	Denies, nuances	I26
	Answers	I27
	Asks for clarification	I29
Content	Child’s perspective	C1
	Perspective taking, analysis of situation	C2
	Culture, personality, interiorization	C3
	Function of observations	C4
	Previous training, foster home	C5
	Teamwork and reflection	C6
	Original research, researchers’ perspective	C7
Paralinguistic	Vocalization to confirm: *Ajam, igan*	P1
	Laughs	P2
	Coughs	P3
	Laughs + vocalization to confirm	P4

Dimensions, sub-dimensions and their corresponding codes.

### 2.3 Procedure

In line with the three macro-stages QUAL-QUAN-QUAL proposed by OM within the mixed methods approach, this study follows the proposal developed by Anguera (2021) to analyze in-depth interviews through indirect observation.

First of all, and once the purpose of this work was formulated, a plan was designed to obtain the required information. We decided to conduct a single interview with the selected participants, with whom training sessions had previously been held and, therefore, were already aware of the results of the original research, “Pikler educators early in the morning.” The interview was carried out following an open script or outline as a general guideline. First of all, the interview was divided into three sections. Each of them referred to one of the central keys found in the original research, which we aimed to discuss with the interviewed educators. As presented in the introduction, these keys are: 1) the stability found in the habitual behavior of the educator; 2) the educator’s focus of attention depending on her aim to intervene or not; 3) the educator’s emotional control during her interventions.

Once the themes of the interview had been established, several questions were asked about each of them with the aim of clarifying, defining and agreeing on their meaning. Thus, these questions were not designed to be formulated literally during the interview, but rather as a resource in case it was necessary to clarify the topic. For example, the first central key was posed as follows: *“previous research has resulted in behavioral patterns that report that educator’s behavior is very stable, because there is a sequence of actions she systematically repeats.”* Next, questions were made: *“do you agree with this characteristic we found within your daily work? Do you have a choreography or protocol that you systematically follow in each activity or body care? Do you always try to do it the same way?”*

The interview was conducted and transcribed, and then this transcription was transferred into Excel, once relevant decisions about how to code the interview had been made. In this sense, the first decision concerned the segmentation of the transcript into textual units. There are several criteria that can be applied: orthographic, syntactic, contextual, interlocutory, etc. This decision is closely related to the pursued degree of molecularity. Specifically, in this work, two of the above-mentioned criteria were adopted and combined: first, the interlocutory, understood as the turns of speech throughout the interview and, second, the orthographic, so each sentence was established as a textual unit. The last step of this qualitative stage involved the systematization of the recording, carried out by assigning each unit of analysis a code or codes within the observation instrument. Given their mutual exclusivity, each unit was assigned as many codes as co-occurrences between the different dimensions included in the instrument were detected.

The second stage, which is predominantly quantitative in nature, started with the development of the systematized recording. This is the step in which *quantitizing* occurs, an important step in research within mixed methods. It is, precisely, the process where the primary parameters of the recording proposed by Bakeman (1978) are obtained: frequency, order and duration. The proposal developed within OM is fundamentally based on order or sequence (Anguera et al., 2017, 2021), that is, the code matrices are formed by successive cooccurrences of codes —the rows of the matrices— which allows the application of various quantitative techniques of analysis appropriate for diachronic data. Before analyzing the data from these code matrices, they must be subjected to quality control, as this methodology undoubtedly requires an effort to detect biases in the recording, as well as mismatches between observers. Therefore, in this case, the three researchers systematically recorded the whole interview, obtaining three different code matrices. There are numerous coefficients to quantitatively verify the quality of data, but in this case, canonical concordance coefficient (Krippendorff, 2013) was chosen, since it permitted to calculate the concordance between the three recordings. This coefficient was calculated using HOISAN and obtained a satisfactory result of 71.99%.

Once the quality of data had been checked, data analyses were carried out. In this case we opted to use a technique particularly suitable to detect structures within relationships between codes: lag sequential analysis (Bakeman, 1978; Bakeman and Quera, 2011).

This technique is used to identify regularities between observed behaviors and, therefore, discover associations between them, specifically, through the calculation of observed and expected probabilities (Bakeman and Quera, 2011). Starting from a certain behavior, known as criterion behavior and selected based on the study objectives, this analysis shows what other behaviors —conditioned behaviors— precede and follow it, through retrospective (lags −1, −2, −3, etc.) and prospective (lags +1, +2, +3, etc.) lag sequential analyses. The results obtained are called adjusted residuals and show the likelihood of appearing of each conditioned behavior in each lag that is studied. Depending on the value of obtained adjusted residuals, it can be concluded if a behavior’s likelihood of appearing in a specific lag is higher than the effects of chance, with a significance level of *p* < 0.05 (adjusted residual >1.96). When the obtained adjusted residual is negative, it shows that the relationship between criterion and conditioned behaviors is inhibitory. Concurrence of behaviors can also be studied, considering adjusted residuals in lag 0. The data used for this analysis were type II: event based and concurrent (Bakeman, 1978), and data analyses were performed in SDIS-GSEQ v. 5.1 (Bakeman and Quera, 2011).

In order to satisfy objective 1, we performed a lag sequential analysis to check for a potential communicative pattern within participants’ discourse when answering to the proposed questions. For this analysis, we used the recording of the researcher who had the leading role in this study. When it comes to analyses responding to objective 2, we explored concurrences between behaviors and, in this case, we performed the same analyses with the recordings made by the three researchers, aiming to reach more conclusive results.

After the quantitative results are obtained, these have to be interpreted. The return to a qualitative phase in the study of the in-depth interview closes the circle of the connect option (Creswell and Plano Clark, 2011), situated within mixed methods.

## 3 Results

The first objective of this research was to detect if the interviewed educators, who count with a long experience working under the principles of Pikler-Lóczy education, confirmed the existence of the three central keys found in our original research and if, in addition, they recognized them as their own.

In order to achieve this objective, a lag sequential analysis was carried out. Among the behaviors included within the communicative intention dimension of the field format, “interviewer asks” (I11) was introduced as a given behavior and all those related to the interviewees’ communicative intention were selected as target behaviors: confirms (I21), adds new information (I22), exemplifies (I23), reformulates in the same sense (I24), reformulates in another sense (I25), denies/nuances (I26), answers (I27) and asks for clarification (I29).

**Figure 1:**
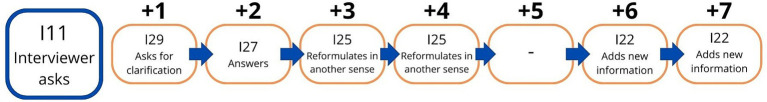
Communicative pattern within the interview.

Positive lags, that is, those belonging to the prospective perspective of lag sequential analysis, were analyzed. Significant adjusted residuals found in each lag are included in Table 2 and result in a pattern shown in Figure 1.

**Table 2:** Lag sequential analysis of communicative intention.

	Lag +1	Lag +2	Lag +3	Lag +4	Lag +5	Lag +6	Lag +7	Lag +8	Lag +9	Lag +10
I21 – Confirms										
I22 – Adds new information						**2,336**	**3,107**			
I23 – Exemplifies										
I24 – Reformulates in the same sense										
I25 – Reformulates in another sense			**2,024**	**2,016**						**1,97**
I26 – Denies/nuances										
I27 – Answers		**2,609**								**3,684**
I29 – Asks for clarification	**3,573**									

I11 (interviewer asks) as given and interviewees’ communicative intention behaviors as target. Significant adjusted residuals on positive lags. Bold values significant adjusted residuals in each of the studied lags.

Specifically, when the interviewer asks educators a question, they initially respond with another question (I29) that seeks to clarify the discussed topic. Then, the interviewee answers (I27) directly to that question. After this, she reformulates in another sense (I25) in two consecutive lags, which would add nuance to her argument. In the fifth prospective lag, no significant behaviors were found, potentially indicating that the interviewer adds another question or intervenes to clarify something that is being discussed. This idea is reinforced by the behaviors observed in the following two lags: in both of them the interviewee adds new information about the topic they are talking about (I22). As no significant behaviors are found in the next two lags, this would constitute the end of the behavioral pattern.

The second objective of this work was oriented to reveal new theoretical-practical elements that could could broaden or enrich, broaden or enrich the interpretation of the Piklerian educational practice. In this case, we applied the same technique, lag sequential analysis, but just to analyze concurrences. Specifically, we studied concurrences between the three central keys found in our original research —stability (A1), focus of attention and strategic positioning (A2), emotional control (A3)— and the aspects related to the content of the interview that were found when developing the indirect observation instrument: child’s perspective (C1); perspective taking/analysis of the situation (C2); culture, personality, internalization (C3); role of observation (C4); previous training in the foster home (C5); teamwork and reflection (C6); original research and researchers’ perspective (C7).

Given that the three observers performed a systematic recording, the same analysis was conducted with the three samples. Obtained results are presented in Table 3. Regarding to stability (A1), concurrences were found with the child’s perspective (C1, obs 3), with the analysis of the situation (C2, obs 2) and with aspects related to our original research (C7, obs 1, 2 and 3). In addition to these concurrences, we found a significant negative adjusted residual in one case: culture (C3, obs 2 and 3).

**Table 3:** Comparison of adjusted residuals obtained by analyzing the systematic recording of each of the three observers.

	A1			A2			A3		
	OBS1	OBS2	OBS3	OBS1	OBS2	OBS3	OBS1	OBS2	OBS3
C1		3,02	2,53	4,37	3,58		−3,61	-3,31	3,42
C2					4,45			−6,35	
C3		−2,74	−2,36	−2,93	−4,6		3,84	6,29	
C4						4,23			−3,35
C5					−2,19			3,25	2,75
C6					−2,32	−3,02	1,99	3,17	4,16
C7	4,26	4,85	5,87			−2,33		−2,11	−2,04

Significant concurrences between the three central keys and the seven aspects of the interview content.

As for strategic positioning (A2), we found concurrences with the following content aspects: child’s perspective (C1, obs 1 and 2), analysis of the situation analysis of the situation (C2, obs 2) and function of observations (C4, obs 3). Significant negative adjusted residuals were also found with culture (C3, obs 1 and 2), previous training in the foster home (C5, obs 2) and teamwork (C6, obs 2 and 3).

Finally, when analyzing emotional control (A3), concurrences were found with culture (C3, obs 1 and 2), previous training in the foster home (C5, obs 2 and 3) and teamwork (C6, obs 1, 2 and 3). Significant negative adjusted residuals were also found: child’s perspective (C1, obs 1, 2 and 3), function of observations (C4, obs 3) and original research (C7, obs 2 and 3).

## 4 Discussion

The general objective of this study has been met since it was possible to contrast the central results obtained in the original research “Pikler educators early in the morning” by means of an in-depth interview (Anguera, 2021) —a modality of indirect observation within mixed methods— with the two educators observed. The results of the original research were demonstrated as central keys of the Pikler-Lóczy educational approach. In the first place, we found the always adaptable stability of the educator’s behavior to the human and physical circumstances of each moment or educational situation. Thus, depending on the child or children she is intervening with and the classroom space she is in, the educator’s behavior is extremely stable, almost choreographic and performative, so that she emanates a fundamental security for an optimal child development. Secondly, the educator always shows an interested positioning. It is never casual, but instead, very conscious and intentional. If she is not present or nearby, she observes attentively or seeks information verbally or using gestures. This positioning is strategic, so the educator’s focus of attention is always optimal; this is especially beneficial as attention in a collective environment is necessarily varied, and it also contributes to an ergonomic educational management. Third, there is an active and fluid emotional control, typical of conscious professional empathy, so the educator is known to be a permanent role model, who yields the protagonism of their education to babies and young children themselves. This aspect makes the studied educational approach truly *paidocentric*.

The first specific objective was confirmed as we obtained a defined communicative pattern. The three central keys discussed throughout the interview were not denied —there is no denial (I26) in the pattern—, so they would be confirmed; however, they were not confirmed as initially presented by the interviewer. Instead, interviewed educators reworked those ideas their own way, as shown in the obtained behavioral pattern. Educators do so through questions to clarify (I29) the presented idea, some punctual answers (I27), reiterated reformulations (I25) and the contribution of new information (I22) because they evidently know the details of their own activity better than the interviewers. In other words, they use the typical Piklerian model of shared reflection, always dialectical and open to nuance, which has developed since the very origin of the educational model, and which has made the educators the professionals they are. Evidently, while doing so they seek to ensure that the terminology is common and that the concepts and vocabulary used are well defined and shared by both interviewees and interviewers. This makes it possible to establish better comparisons and analyses and optimizes both their observational modality and their educational model.

The second objective, on the other hand, was refuted. Despite recognizing the three central keys as their own, albeit with nuances, the obtained results do not point to new Piklerian theoretical-practical elements to contribute. They limit themselves to confirming certain central issues of the model. Thus, stability (A1) takes into account the child’s perspective (C1), as well as the situation (C2) in which the activity takes place, and is also related to the research findings (C7) explained to them. At the same time, this central key of Piklerian education seems to be something structural or part of the model itself and not the result of internalization or acquired culture about children (C3). Indeed, the ability to give stability to each moment and, with it, the adaptability to children flows in their daily lives, it is not an idea or a concept that is reached, but a permanent educational exercise with specific children in the concrete situation in which he/she finds him/herself, which was precisely the object of the original research that was being contrasted in the interview. Something similar happens with strategic positioning (A2). It is again related to the child’s perspective (C1), to the analysis of the specific situation (C2), as well as to the observation of the context (C4). This means that the educator’s positioning is never a result of chance, but she focuses on the child she is intervening with and the context. Culture (C3), previous training in the foster home (C5) and the educational team’s continuous reflections (C6) are absent when analyzing their relationship with strategic positioning (A2). Finally, when it comes to emotional control (A3) there is a certain inversion compared to the previous aspects, since it does seem to be associated with culture (C3), previous training in the foster home (C5) and the educational team’s continuous reflections (C6). In other words, it seems that emotionality would be a factor that requires permanent personal reflection among educators, which the two previous keys —stability (A1) and strategic positioning (A2)— do not require, since they would flow without any effort in educators’ usual intervention. Moreover, it would be an intrinsic and generalized emotional control, not associated to the specific child (C1), nor to the specific situation (C2) in which he/she finds him/herself, nor to the observation of the context (C4). Neither is it related to the original research (C7), although it should, since it is one of its vertebral results. All in all, it seems that the perspective of the interviewed educators is that “they are able to do Piklerian style”, which would be understandable after so much training, professional experience and shared reflection.

The presented methodological proposal can be considered a novel approach to mixed methods, since the possibilities offered by indirect observation to analyze the conducted in-depth interview are evident. The potential of this study lies, precisely, in the simplicity of the equipment used, since an austere 47 minute in-depth interview and the application of an exclusive analysis technique —lag sequential analysis— led to generous results and conclusions. Thanks to lag sequential analysis in its positive perspective, a stable response pattern from the interviewed educators has been obtained, a faithful reflection of the Piklerian modus operandi guided by the fact that “knowing the child” is a necessary condition to be able to help him/her. It is not always enough, but the team always goes back to the question —and does it as many times as necessary— until a satisfactory and adjusted answer is found. Regarding the analysis of concurrences, it unraveled certain relationships between previously obtained results, although it basically confirmed that the Piklerian intervention style, once internalized, is not understood as such, but is part of the educator herself, with the exception of emotions, which must be kept under control on a daily basis.

In summary, this work demonstrates the perfect fit between observational methodology and Pikler-Lóczy education once again. Moreover, it has allowed us to confirm some of the results obtained through direct observation in our original research. This supports the convenience of the application and conceptualization of mixed methods, while opening the door to a more exhaustive investigation to contrast other results obtained in our original research.

## Data availability statement

The raw data supporting the conclusions of this article will be made available by the authors, without undue reservation.

## Ethics statement

The studies involving humans were approved by the Ethics Committee of the University of the Basque Country (UPV/EHU). The studies were conducted in accordance with the local legislation and institutional requirements. The participants provided their written informed consent to participate in this study.

## Author contributions

HB: Data curation, Formal analysis, Methodology, Writing – original draft. JS: Conceptualization, Investigation, Writing – original draft. EH: Conceptualization, Supervision, Writing – original draft.
